# Abstract of the Proceedings of the Buffalo Medical Association

**Published:** 1862-09

**Authors:** J. F. Miner

**Affiliations:** Secretary


ART. II.—*Abstract of the Proceedings of the Buffalo Medical Association.*

TUESDAY EVENING, August 5th, 1862.

Present—Prof. James P. White, President, in the Chair; Drs. Samo, Ring, Eastman, Congar, Rochester, Shaw, Wyckoff, Miner.

*Voted*, That Dr. McKinnon be admitted a member upon compliance with the By-Laws.

*Dr. Rochester* said, that for the purpose of calling attention to the subject, he desired to make a remark upon measles, which had been and still is prevailing to some extent. Most cases observed are natural and regular in their usual features, while in some cases patients have been quite ill for ten or twelve days, with fever coming on slowly, headache, pains in the back and limbs, with not much injection of the eyes, coriza, or cough, when an eruption like rubeola would appear extending over the surface. One patient had been left in his care by Dr. White, presenting these symptoms. He had then a patient just recovering from a severe attack. A child in the family had measles; in two weeks the mother commenced to complain of pain in the head, back, &c., with chill, and was very ill for ten or twelve days, when eruption as described appeared, the exact character of which he could not determine.

*Dr. Miner* had observed a case of eruptive disease somewhat anomalous in character which he desired to relate in this connection. A young woman had been ill for three or four weeks, but not obliged to keep her room. At length she had headache, chill, pains in the back and bones, and fever; this was finally attended by an eruption somewhat like measles. The elevations were smaller than usual in the eruption of measles, and a universal redness resembling scarlet rash was present, extending over the entire surface. Five weeks previous to this attack the lady's little child had scarlet fever. Two weeks after this attack her sister had disease similar to herself, and a few days later another sister suffered the same way. One of these sisters was visited by Dr. Rochester, who, as he had been informed, called the disease roseola, after observing it for a day or two, at first, being unable to decide that it was not measles. The glands upon the back of the neck were enlarged, and remained so for several days after the disappearance of eruption. The eruption sufficiently resembled roseola as described by authors upon eruptive diseases, but the evidences of communicability being somewhat conclusive, and the distinguishing characteristic of roseola as given by Willan, Wood, Watson and others, being that it is not contagious. had continued doubt in his own mind as to the nature of the disease.

It should perhaps be said, that there was no cough, injection of the eyes, or other symptom of measles in the case he had seen other than the eruption, and he should have had no hesitation at any time in saying it was not measles. At the first, he regarded the eruption as a rash, produced by some derangement of the stomach, from indigestible food, or other cause which he could not trace, until the occurrence of the same disease in the sisters who had been clearly exposed. After being in possession of all the facts, and considering the case fully he would only express doubts as to the nature of the disease, and be glad to see any light which could be thrown upon it.

*Dr. Rochester* remarked that he recollected the case referred to by Dr. Miner. That he thought at first it was measles. Did not know that the diagnosis could be always made out. One form, of Roseala, Roseola-Dentata, had been regarded as a variety of measles, and contagious in character. Wilson in his work on skin diseases was referred to as taking this view. Still, this might not be correct. Dr. J. C. Peters, the editor of the *Homeopathic Medical Journal*, who has recently published his renunciation of Homeopathy, in an early number of his journal, speaking of roseola, as prevailing in New York during the winter of 1859–60, says: "By many physicians it was mistaken for measles; in fact, in most cases, the resemblance was so strong to 'rubeola sine catarrho' that many, relying upon the epidemic prevalence of rubeola, decreed almost all eruptive affections to be cases of this disease; numerous others mistook the affection for a hybrid of measles and scarlatina. In many cases, although the eruption was abundant, the patients were able to be up, and the whole affection disappeared in two or three days; in other instances it lasted for a week or ten days. Any one who has seen much of roseola will agree with Wilson that it is distinguished from other exanthemata by *negative*, rather than positive characters; and that the diseases with which it is most likely to be confounded are rubeola, erythema, urticaria, purpura, and scarlatina. Although non-contagious and non-infectious, still the epidemic influence may be so great that many are attacked in the same house; and I have seen a new-born babe take it a week after its mother had recovered from it. In general appearance the rash resembles rubeola, but, on closer examination, is found to consist of patches of larger size and more irregular form; and, at a later period, the difference is still more striking, in consequence of the change of tint to a dark roseate hue, which may be followed by petechial or ecchymosed spots and a discoloration like that which follows a bruise. Although there is generally little or no cough, still occasionally there is some, and more or less redness of the fauces."

*Dr. Congar* said that twelve years since he came to a conclusion; does not know whether it is right or not. When called to a patient in the early stage of eruptive disease, previous to eruption, if the pulse is rapid, 120 per minute, he concludes it will have scarlet fever. If the pulse is not over 90 per minute the disease will prove to be measles. In this diagnostic symptom he places great confidence; has never been disappointed, and is always able to predict from this symptom alone the nature of the disease, if it prove to be either measles or scarlet fever.

*Dr. Eastman* remembers having treated cases like those described by Dr. Rochester. Did not regard the disease as measles from the commencement, but as appearing upon the illness which had preceded. Related a case, sick as had been described for ten or twelve days, for which he had prescribed quinæ. When passing he called as a complimentary visit, and found measles fully developed. He also spoke of recently finding varicella, rubeola and scarlatina, in the same district,. all running a natural course. Had some doubts as to the character of the varicella at first. Has never observed the frequency of the pulse as indicating very positively the appearing disease, as has been remarked by Dr. Congar. Has seen patients with as frequent pulse in the early stage of measles as in scarlet fever.

*Dr. Wyckoff* enquired if measles was often observed to appear the second time? had not often heard of well authenticated cases. Had recently known of one patient who he had no doubt had measles the second time; the second attack six or seven weeks after the first. Had never seen such a case before.

*Dr. Eastman* had seen one case which was well marked and unmistakable measles, and it was said by very intelligent parents to have had the disease before, equally well marked and unmistakable.

*Dr. Rochester* thought, that there are well authenticated cases of measles appearing the second time; cases are quite common, *said* to have it the second time. Dr. R. related the particulars of a case which he visited while a young physician, practicing in New York City, and diagnosed as measles. He was soon called upon by the father of the patient, who was a crusty, obstinate old gentleman, and asked for his bill, which was paid, and he was informed that he need not continue his attendance, for "children don't have measles the second time." Crusty old gentleman called in a day or two and again desired his attendance, satisfied that the child had measles, whatever it might have had previously.

*Dr. White* remarked upon the tardiness of the appearance of the eruption in many cases of measles he had seen, but had observed nothing else remarkable in the progress of the disease. The recurrence of small pox, scarlet fever, and other eruptive diseases, is fully established, and he had no doubt but measles did occasionally appear the second time.

*Dr. White* reported the following case of cerebral disease, remarking upon the great obscurity which is conceded to exist in many if not all diseases of the brain:

Robert ——, aged 16 years, of general, robust appearance and good health, came home from a visit to Niagara, complaining of most terrific pain in the head, and in the whole system. Could not always locate the pain. Said it was "all over." Pain was very violent, and at times attended by paroxysms, when he seemed in perfect agony. The first visit was made about the middle of April, and quinine and morphine were prescribed. Attention was early directed to the head, and though the case was very obscure, still there was evidence of cerebral disease sufficient to fix the impression quite strongly. Pupils natural, no paralysis, would take medicine, protrude the tongue and answer questions when asked; would tell, or try to tell, how he felt. Was visited, during his absence for a few days, by Drs. Rochester and Eastman, who also regarded the disease as probably cerebral. The pulse was slow from the commencement; this was what first led him to mistrust cerebral disease. Early in July stomach became irritable, and food was not retained; enemata were resorted to, to sustain the patient. Emaciation became extreme. The wonderful intellectual clearness, slowness of the pulse, and absence of paralysis are remarkable phenomena in connection with the disease found upon examination after death.

*Post mortem* examination by Dr. Babcock and Mr. Tefft. Present with himself, Drs. Rochester, Eastman and Miner. Great emaciation; injection of piamata; ventricles distended with sero-purulent infiltration; cerebellum softened and completely broken down. Other organs not examined.

That this amount of disease and disorganization could have been present and life still be continued, seemed quite remarkable. The boy was able to say prayers with the family the day before his death, and no great change had been noticed in his condition for several days. That the functions of the brain could have been continued in any degree with this amount of organic change, is the remarbable feature of the case.

*Dr. Rochester* remarked that when he first saw the patient the disease was not clearly in the brain; indeed had the stomach been found to have been the principal seat of the disease no one who had observed the case would have been much surprised. He believed with Dr. White from the first that the disease was probably in the brain.

Dr. R. referred to a case he had recently treated of acute hydrocephalus. Patient was attacked with great pain and paralysis, but the mind was perfect. On *post mortem* examination a pint of serum was found in the ventricles. Brain in this instance was hardened, eyes protruded from pressure within; and yet the intellectual faculties were unimpaired.

*Dr. White* remarked upon the value of Oxalate of Cereum as a remedy for the sickness of pregnancy. Had found it recommended in Simpson's Notes, and used it, in some cases, with most signal success. Generally prescribed it to be taken in two or three grain doses after each meal. It seemed to act as a sedative to gastric irritation. While perhaps an improvement might in some instances be made by combining with it sub. nit. bismuth and carbonate of magnesia, still he had obtained from its use alone, more happy results than from any other remedy. As yet he had had, but limited experience, not having prescribed it in but few instances, but in these few, with the happiest effects.

*Dr. Rochester* had prescribed the Oxalate of Cereum in five cases; in four out of the five it had been successful. Prescribed one grain three or four times, daily. In one case it answered well for a few days, and after that was ineffectual. One case the patient was very ill; had been under Homœophathic treatment. Had taken all sorts of remedies—Oxide of bismuth—Iron in various forms—Arsenic—Hydrocyanic acid, &c., &c. He gave Oxalate of Cereum which was quite effectual, after many other remedies had failed.

*Dr. Congar* spoke of the value of hygienic measures in the treatment of such cases, and particularly of the importance of not eating rapidly, and leaving off while hunger yet remained. Had known cases relieved by attention to diet, when all remedies seemed inefficacious.

*Voted* to adjourn to the first Tuesday evening in September.

J. F. Miner, Secretary.

